# Analysis of Force Signals for the Estimation of Surface Roughness during Robot-Assisted Polishing

**DOI:** 10.3390/ma11081438

**Published:** 2018-08-15

**Authors:** Beatriz de Agustina, Marta María Marín, Roberto Teti, Eva María Rubio

**Affiliations:** 1 Department of Manufacturing Engineering, Universidad Nacional de Educación a Distancia (UNED), C/Juan del Rosal 12, E28040 Madrid, Spain; mmarin@ind.uned.es (M.M.M.); erubio@ind.uned.es (E.M.R.); 2 Department of Chemical, Materials and Industrial Production Engineering, University of Naples Federico II, Piazzale Tecchio, 80, 80125 Naples, Italy; roberto.teti@unina.it

**Keywords:** robot-assisted polishing, force signal, surface roughness, end point detection

## Abstract

In this study feature extraction of force signals detected during robot-assisted polishing processes was carried out to estimate the surface roughness during the process. The purpose was to collect significant features from the signal that allow the determination of the end point of the polishing process based on surface roughness. For this objective, dry polishing turning tests were performed on a Robot-Assisted Polishing (RAP) machine (STRECON NanoRAP 200) during three polishing sessions, using the same polishing conditions. Along the tests, force signals were acquired and offline surface roughness measurements were taken at the end of each polishing session. As a main conclusion, it can be affirmed, regarding the force signal, that features extracted from both time and frequency domains are valuable data for the estimation of surface roughness.

## 1. Introduction

These days, finishing processes such as polishing are not yet fully automated in manufacturing industries. Traditionally, polishing has largely been a manual operation that is very labor-intensive, highly skill-dependent, inefficient due to long processing times, high-cost, error-prone, and hazardous due to abrasive dust [1]. For this reason, a considerable percent of the total time required for manufacturing products such as dies, molds, machine tools, and optical components is spent on the finishing operations, which account for approximately 30% to 50% of the manufacturing time [2,3].

Due to the fact that polishing is not performed by position control but by pressure control, efforts towards automated polishing have mainly focused on the identification and the execution of the motions involved in manual polishing techniques, performing under a desired contact force exerted on the tool [4]. This is an important challenge in die polishing, especially for the manufacturing of components with complex shapes and machine tools that contain freeform surfaces and functional relevant edges.

The automated polishing systems developed until now can employ a conventional machine tool structure or an articulated robot arm to hold the finishing tool. For both configurations, force control methods have been designed and can be broadly classified as active and passive force controls. Active force control systems measure and correct the force applied on the workpiece, independently of the tool path, and passive control systems rely on compliance in the tool itself to maintain a nominal contact force or rely on magnetic force to achieve this [1,5,6,7].

Also, the polishing tool-path has been controlled by the integration of intelligent network systems, using multiple vision sensors to capture images of the polished surface as input collected data [8]. Furthermore, tool-path planning methods based on contact mechanics have been designed for automated polishing [9,10,11].

Apart from the approaches made towards the tool-path planning for automated polishing processes, there are other studies focused on endpoint detection; this occurs when a certain target surface roughness value is reached, or it is required to replace the tool with another one with finer grain size and/or implement other polishing conditions. As a result, the polishing sequence can be improved. For this purpose, signals from different sensors have been employed to monitor the surface roughness obtained by polishing. In this scheme, signals from AE, motor current, and light sensors have been employed for the development of expert systems integrated with the data collected during the polishing process [2,6,12].

In this study, feature extraction of force signals detected during robot-assisted polishing processes was carried out to estimate the surface roughness during the process. The purpose was to collect significant features from the signal that allow the determination of the end point of the polishing process based on surface roughness. For this objective, dry polishing turning tests were performed on a Robot-Assisted Polishing (RAP) machine (STRECON NanoRAP 200) during three polishing sessions, using the same polishing conditions. During the tests, force signals were acquired and offline surface roughness measurements were taken at the end of each polishing session.

## 2. Experimental Procedure

The experimental procedure of this study as follows.

### 2.1. Polishing Tests

**Figure 1:**
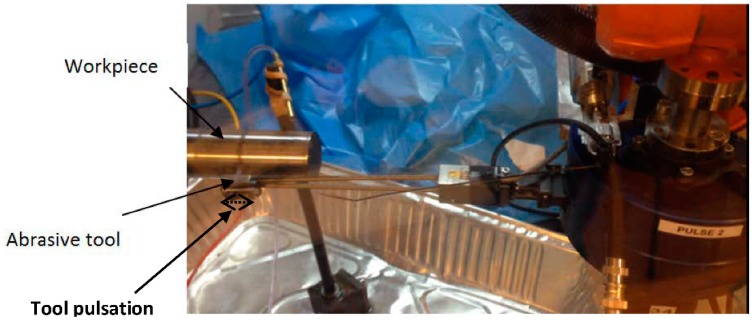
Robot-Assisted Polishing (RAP) machine.

The polishing tests were performed by a Robot-Assisted Polishing (RAP) system (STRECON NanoRAP 200, Sønderborg, Denmark) fitted with a robotic arm in which interchangeable polishing tools are mounted. Four cutting parameters can be set up by the integration of a control module in the robotic arm: cutting speed, feed rate, the contact force between the tool and workpiece, and the tool pulsation in the feed direction. Three sessions of polishing tests (60 polishing passes each one) were carried out on a cylindrical probe of alloy steel (UNS G52986) with a length of 75 mm and diameter of 30 mm (Figure 1). The same cutting parameters were employed for all polishing sessions: spindle speed of 300 rpm, feed rate of 5 mm/s, tool pulsation in the feed direction of 500 pulses/min on a length of 1 mm and a contact force of 9.8 N (movement indicated in Figure 1). The abrasive tool selected has a grit number of 800, which corresponds to a grain size of approximately 11 μm from Gesswein (MP800) according to the Commercial Standard CS271-65 [13].

### 2.2. Acquisition of Force Signal during Polishing Tests

A gauge was employed to detect the force signal during the polishing tests. First of all, a calibration process was made to establish a relationship between the voltage signal generated on the gauge device and the corresponding force. Table 1 shows the different forces (N) used and the voltage signal recorded. For the polishing tests, signal samples were taken with an interval time between acquisitions of, approximately, 0.93 s. That is, across the three polishing sessions (180 passes), there was a total of 2790 acquisitions, with 16,384 data points for each acquisition. The sample rate was set up for 50 kHz.

At this point, it is important to remark that the signal force plotted on the different figures along the present paper is expressed in volts; the objective is to show a trend of the values of forces rather than calculating their absolute values.

### 2.3. Surface Roughness Measurements

Five offline surface roughness measurements were taken on the workpiece (on one of its generatrix) at the end of the each polishing session, using a roughness MahrSurf XD1 (Mahr, Göttingen, Germany) tester equipped with a radius tip of 2 µm. The cut-off selected was 0.25 mm and the evaluation length was 1.25 mm. In the measurement process, data (x_i_, z_i_) of the surface geometry workpiece were registered for the determination of the arithmetical average roughness parameter, *Ra*. According to ISO 4288 (ISO 4288, 1996) [14] standards, such a parameter is defined as the arithmetical average of the absolute values of the deviations of the roughness profile, *R*, and is expressed mathematically by means of Equation (1):

(1) $$Ra=\frac{1}{l_{m}}\int_{0}^{l_{m}}\left|z(x)\right|dx.$$

### 2.4. Analysis of Force Data

Row force signal data arrays, each with 16,384 values, are stored and analyzed by Matlab software (R2011b, Mathworks, Natick, MA, USA). Different features were calculated from both the time and frequency domains. In the time domain, a preliminary analysis was made. Different statistical features were calculated from the 2790 signal acquisitions and plotted for each feature. For those feature plots in which a trend can be clearly observed, the average and variance were also determined for each polishing session at the last three passes.

In the frequency domain, *FFT* (Fast Fourier Transform) power spectral graphics at different polishing time were plotted. The objective is to select a range or ranges of frequencies in which the amplitudes of certain peaks shown in their corresponding *FFT* power spectral plots may change over time, just as the AE (Acoustic Emission) signal detected during polishing has been processed in previous studies [15,16,17].

Once the range of frequencies was selected, the maximum amplitude was calculated from all the force signal acquisitions registered. Afterwards, the average and variance were also calculated for each polishing session at the last three passes.

Finally, an analysis of the features extracted from the time and frequency was carried out to identify a possible trend and a correlation with the surface roughness.

## 3. Results and Discussion

**Figure 2:**
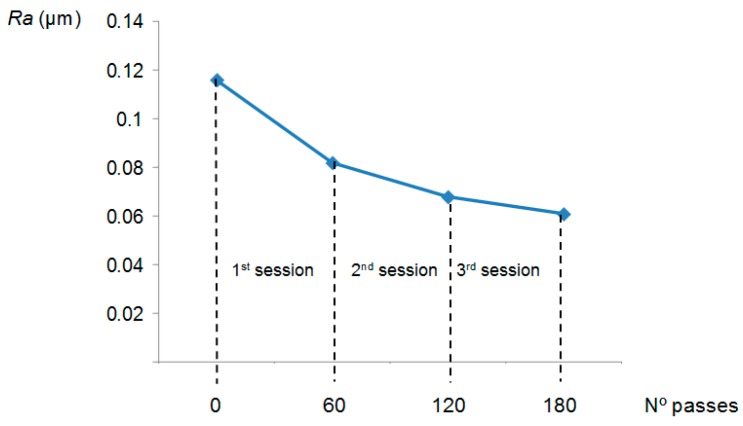
*Ra* versus polishing passes obtained at the end of each polishing session.

The resultant *Ra* calculated for each polishing session, together with the surface roughness measured before polishing, is represented in the Figure 2.

**Figure 3:**
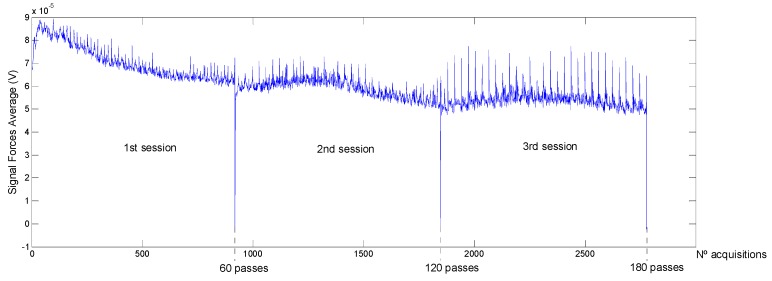
Signal forces average (*F_average_*) during the three polishing sessions.

**Figure 4:**
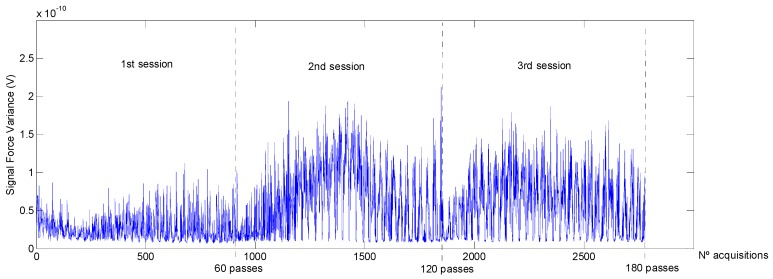
Signal forces variance (*F_variance_*) during the three polishing sessions.

As was expected, *Ra* decreased with the number of passes. In a first step (1st polishing session) the decrease is more accentuated due to the higher quantity of material removed in the first stage of polishing. Hereafter, the analysis of the different features extracted from the force signal is shown and discussed. At time domain, among all the statistical features calculated from the row force signal, only average and variance follow a trend with polishing time, designated in this study as *F_average_* and *F_variance_*. This can be observed in Figure 3 and Figure 4.

Moreover, the fact that *Ra* hardly decreased during the last session (3rd polishing session), from 0.068 to 0.061 µm, indicates that endpoint detection was almost reached. Also, this can be expected as the force signal in the last session presents a periodical form that does not vary with time (Figure 3).

**Figure 5:**
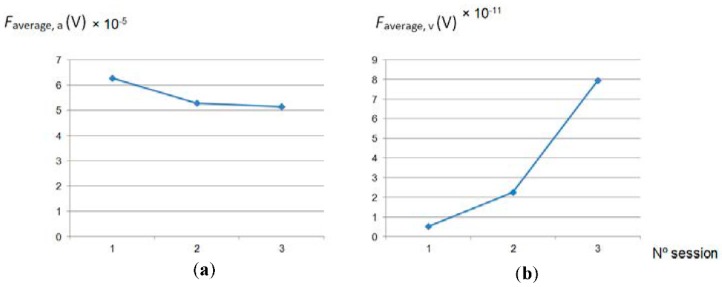
Signal: (**a**) arithmetical average and (**b**) variance, calculated from signal forces average versus number polishing session.

**Figure 6:**
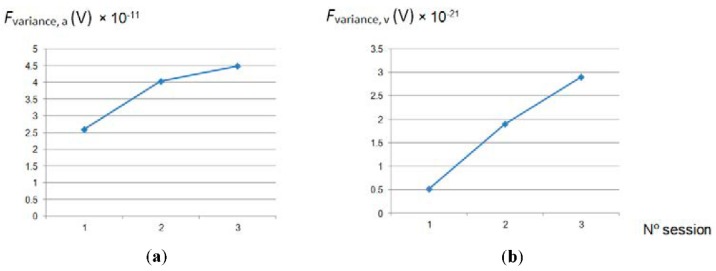
(**a**) Arithmetical average and (**b**) variance, calculated from signal forces variance versus number polishing session.

The trend followed by *F_average_*_,*a*_ (Figure 5a) is the same as *Ra*; it decreases with the number of passes, to a major extent, at the beginning of the process, which indicates that this parameter extracted from the force signal could be used for the estimation of *Ra* during the polishing process. In addition, as was previously pointed out, the quantity of material removed or metal removal rate (*MRR*) decreases with the polishing passes. This parameter also has a direct relationship with the contact force according to the following expression [18]:

(2) $$MRR=\frac{2kr_{avg}Fs}{H_{w}fA},$$

where *k* is a constant *r_avg_*, the average size of the grain tool, *F* the contact force, *s*, the rotational speed, *f*, the feed rate, *H_w_*, the workpiece hardness, and *A* the contact area between the tool and the workpiece. In spite of the fact that, for all the polishing tests, a constant contact force was applied, contact force and its variations detected along the tests, represented by the parameter *F_average_*_,*a*_, follow the same trend that the *Ra* values obtained. Regarding the rest of the parameters calculated, *F_average_*_,*v*_, *F_variance_*_,*a*_ and *F_variance_*_,*v*_, it can be seen that their tendency is inversely proportional to *Ra* (Figure 5 and Figure 6).

**Figure 7:**
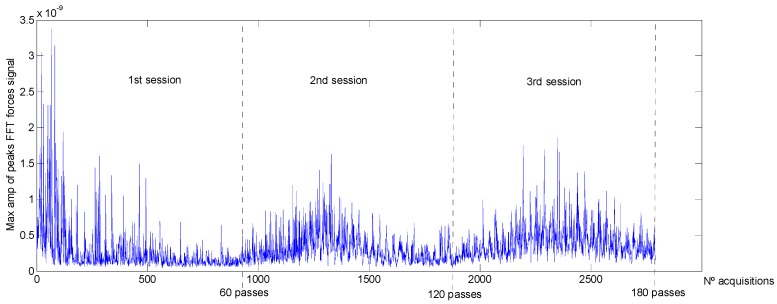
Maximum amplitudes of *FFT* power spectral of forces signal (100–1000 Hz).

From the analysis on frequency domain, according to the peaks shown in the *FFT* power spectral representations, the range selected was between 100 and 1000 Hz. Maximum amplitudes calculated from each acquisition along the three polishing sessions are plotted in Figure 7.

**Figure 8:**
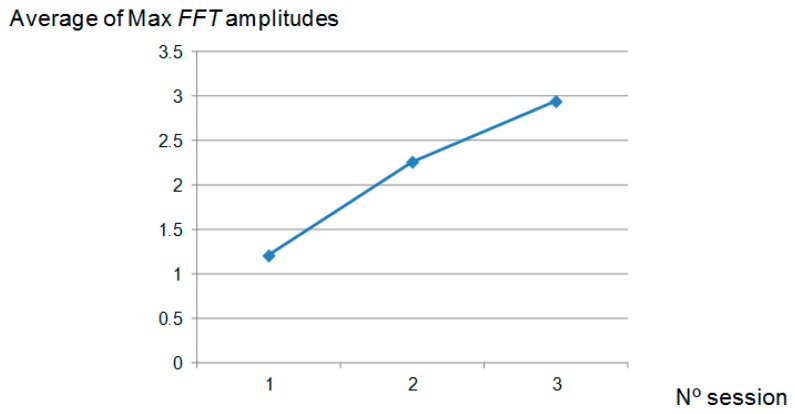
Arithmetical average calculated from maximum amplitudes of *FFT* power spectral of forces signal versus number of polishing session.

Finally, the average of the amplitudes *FFT* power spectral was calculated for each polishing session and shown in Figure 8. It can be seen that the relationship with *Ra* is inverse (see Figure 2) and therefore, it can be considered a valuable parameter for the monitoring of the surface roughness. Nevertheless, this dependency varied within the polishing session: during the 1st polishing session, the parameter decreased with the number of passes and, during the other two sessions, the graphic exhibits a maximum around the middle of the polishing session (Figure 7). Taking this into account, the implementation of this parameter as input data to predict the surface roughness must be considered, together with the number polishing passes.

## 4. Conclusions

In this work, an evaluation of the signal forces detected during Robot-Assisted Polishing (RAP) by a STRECON NanoRAP 200machine was studied. Dry polishing turning tests, under the same cutting conditions, were performed on a cylindrical probe of alloy (UNS G52986) using a tool with a grit number of 800. This semiautomatic polishing system allows the setting of not only conventional polishing parameters such as cutting speed and feed rate, but also the contact force between the tool and workpiece and the tool pulsation in the feed direction. It obtained the trends of different features from the force signal and the surface roughness along the polishing processes to evaluate whether the mentioned features are valuable data for the estimation of *Ra*. For this purpose, an analysis of the force signal, in both the time and frequency domains, detected during the process was made with the objective of finding a feature or features sensitive to the variations of the surface roughness (*Ra*) measured at the end of three polishing sessions. The main conclusions extracted from the results obtained in this study can be summarized as follows:

- After 180 polishing passes under the polishing conditions used in this study, the endpoint detection is almost reached; this is after 45 min of polishing under the cutting conditions employed in this study.
- In spite of the fact that for the all the polishing tests a constant contact force between the tool and the workpiece was applied, different features extracted from the signal force detected during the process can be considered valuable data for the indirect evaluation of surface roughness.
- In the time domain, average and variance were calculated for each acquisition of force signal and from these signals a feature per polishing session (average and variance) was extracted to compare with the surface roughness, designated in this study by *F_average_*_,*a*_, *F_average_*_,*v*_, *F_variance_*_,*a*_ and *F_variance_*_,*v*_. This makes their relationship with the surface roughness, in the case of *F_average_*_,*a*_, proportional, and in the case of *F_average_*_,*v*_, *F_variance_*_,*a*_ and *F_variance_*_,*v*_, inversely proportional.
- In the frequency domain, within the range of frequencies between 100 and 1000 Hz a feature was calculated. This is the maximum amplitude of the peak reached in the range of frequencies for each acquisition of force signal. The average calculated per polishing session is inversely proportional to the surface roughness, so it is a valuable feature for the estimation of *Ra* in polishing processes.
- Finally, for future research it is proposed to collect valuable data from the force signal (concretely average feature according to this study) during polishing processes, together with features extracted from other sensors, to further develop polishing systems.

## Figures and Tables

**Table 1:** Values of forces and the corresponding voltage signal generated during the calibration.

Forces (N)	Voltage Signals (V)
0.0	0.00025
0.98	0.0035
1.96	0.0071
2.94	0.011
4.9	0.0185
6.86	0.0263
